# Transcriptome profiling of bovine inner cell mass and trophectoderm derived from in vivo generated blastocysts

**DOI:** 10.1186/s12861-015-0096-3

**Published:** 2015-12-18

**Authors:** S. M. Hosseini, I. Dufort, J. Caballero, F. Moulavi, H. R. Ghanaei, M. A. Sirard

**Affiliations:** Department of Reproductive Biotechnology, Reproductive Biomedicine Research Center, Royan Institute for Biotechnology, ACECR, Isfahan, Iran; Centre de Recherche en Biologie de la Reproduction, Faculté des Sciences de l’Agriculture et de l’Alimentation, Département des Sciences Animales, Pavillon INAF, Université Laval, Québec, QC G1V 0A6 Canada

**Keywords:** Bovine, In vivo blastocyst, Inner cell mass, Trophectoderm, Transcriptome

## Abstract

**Background:**

This study describes the generation and analysis of the transcriptional profile of bovine inner cell mass (ICM) and trophectoderm (TE), obtained from in vivo developed embryos by using a bovine-embryo specific array (EmbryoGENE) containing 37,238 probes.

**Results:**

A total of 4,689 probes were differentially expressed between ICM and TE, among these, 2,380 and 2,309 probes were upregulated in ICM and TE tissues, respectively (*P* ≤ 0.01, FC ≥ 2.0, FDR: 2.0). Ontological classification of the genes predominantly expressed in ICM emerged a range of functional categories with a preponderance of genes involved in basal and developmental signaling pathways including P53, TGFβ, IL8, mTOR, integrin, ILK, and ELF2 signalings. Cross-referencing of microarray data with two available in vitro studies indicated a marked reduction in ICM vs. TE transcriptional difference following in vitro culture of bovine embryos. Moreover, a great majority of genes that were found to be misregulated following in vitro culture of bovine embryos were known genes involved in epigenetic regulation of pluripotency and cell differentiation including DNMT1, GADD45, CARM1, ELF5 HDAC8, CCNB1, KDM6A, PRDM9, CDX2, ARID3A, IL6, GADD45A, FGFR2, PPP2R2B, and SMARCA2. Cross-species referencing of microarray data revealed substantial divergence between bovine and mouse and human in signaling pathways involved in early lineage specification.

**Conclusions:**

The transcriptional changes occur during ICM and TE lineages specification in bovine is greater than previously understood. Therefore, this array data establishes a standard to evaluate the in vitro imprint on the transcriptome and to hypothesize the cross-species differences that allow in vitro acquisition of pluripotent ICM in human and mice but hinder that process in bovine.

**Electronic supplementary material:**

The online version of this article (doi:10.1186/s12861-015-0096-3) contains supplementary material, which is available to authorized users.

## Background

A distinguishing feature of blastocyst formation in mammals is the specification of the pluripotent inner cell mass (ICM) and multipotent trophectoderm (TE) through a series of highly orchestrated events directed by spatial and temporal modes of gene expression [1]. ICM itself undergoes a second round of cell lineage specification to form the precursors of epiblast (EPI) and hypoblast (or primitive endoderm: PE) [2]. In the mouse, TE gives rise to parts of the placenta and the chorion, the PE develops to parietal and visceral endoderm and the EPI gives rise to the embryo proper, umbilical cords, amnion and part of the chorion [3]. In human and bovine, the PE gives rise to the primitive and secondary yolk sac [2, 3].

It is well-demonstrated that the culture conditions support in vitro capture of mouse pluripotent ICM fail to support human embryonic stem cell (ESC) self-renewal [2]. Ungulates may be a unique case in this respect as none of the current protocols used for in vitro establishment and maintenance of pluripotent cells in the human and mouse embryos have not yet supported the establishment of ESC in none of bovine, ovine, caprine, and porcine species [4]. Therefore, a clear understanding of the gene regulatory networks (GRNs) involved in early lineage specification will explain the difficulty of deriving ESC in mammals other than the rodents and primates, and would illuminate the search for the best functional mammalian model system representative of either early embryo development or stem cell biology [5]. Obviously, analysis of a small number of transcripts is of limited value for the systematic study of genetic interactions in a complex trait, and post genomic area approaches, including genome wide analyses and network investigation, are needed.

Two recent studies examined the transcriptional wiring of bovine ICM and TE tissues derived from in vitro produced (IVP) blastocysts [6, 7]. However, several line of evidence suggests that the initial oocyte quality and post-fertilization culture condition can dramatically alter the transcriptome of embryos compared to in vivo counterparts. For example, Katz-Jaffe et al. [8] demonstrated distinct microarray patterns between bovine oocytes matured in vitro and in vivo, and Tesfaye et al. [9] correlated this transcriptional difference to the distinct transcript abundance of the surrounding cumulus cells. We also previously showed that in bovine, the culture condition during zygote genome activation (ZGA) critically affects gene expression patterns of the resulting blastocysts [10]. In porcine, Withworth et al. [11] observed great transcriptional differences between in vitro- and in vivo-produced embryos. Importantly, Giritharan et al. [12] demonstrated that in vitro culture remarkably reduced the actual transcriptional differences between the ICM and TE tissues in the mouse. Therefore, there is a crucial need to establish a standard transcriptome profile of first lineage segregation in bovine and to evaluate the in vitro imprint on molecular nature of this crucial developmental event.

This study describes the generation and analysis of the transcriptional profile of bovine ICM and TE tissues, obtained from in vivo generated embryos by using a bovine-embryo specific array (EmbryoGENE) containing 37,238 probes [13]. By cross-referencing of the obtained data with microarray data of in vitro bovine ICM and TE, we demonstrated novel information on the effect of IVP on the transcriptional profiles of bovine ICM and TE tissues. Finally, we have collated the transcriptomic data of mouse and human pluripotent cells and contrasted them with our bovine data to distinguish universally applicable and species-specific features of ICM and TE specification across mammals.

## Results

### Embryo recovery data

Production of in vivo embryos was carried out using 26 superovulated Holstein cows and semen prepared from seven bulls as described in the materials and methods section. The superovulation treatment resulted in 4.9 ± 0.9 ovulations per cow and 2.0 ± 0.4 embryos recovered per flush. A total of 277 in vivo generated embryos were retrieved by flushing of at day 7.5 post insemination. Of these, 174 embryos were expanded blastocysts of good quality (Additional file 1: Table S1). Four separate pools of ICM and TE for each treatment were obtained using these selected blastocysts.

### Overview of microarray results

**Fig. 1:**
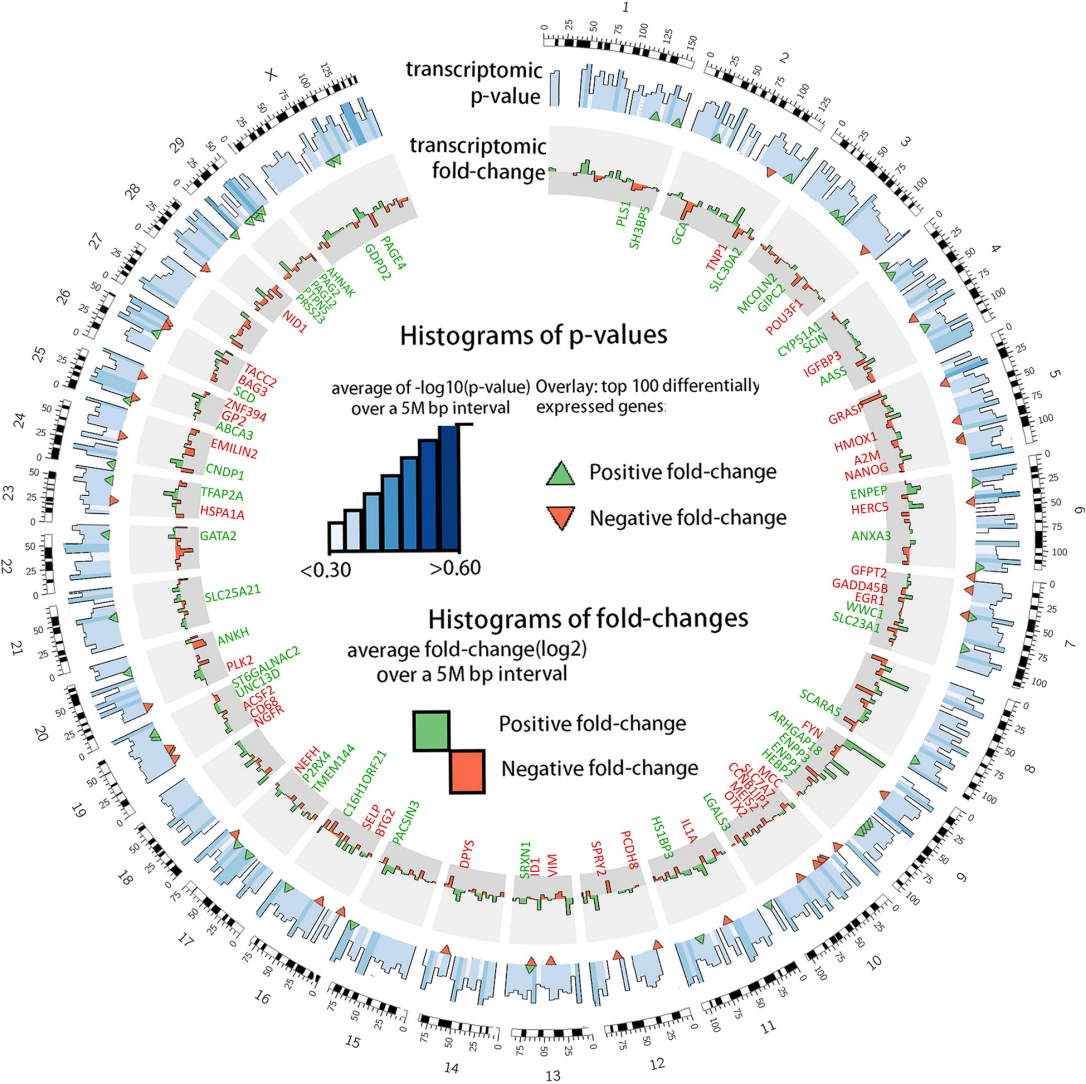
The Circos plot showing the genome-wide transcriptional profiling of ICM and TE obtained from in vivo developed blastocysts. The mean p-values of 5 M bp windows are displayed along with the 100 most significant DMRs. Green overlays in the p-value layer represent upregulated transcripts in ICM, while red overlays represent upregulated genes in the TE. The red and green gene names in the inner layer represent the name of the 100 most differentially expressed genes along with their estimated location in the bovine genome, by chromosome

**Fig. 2:**
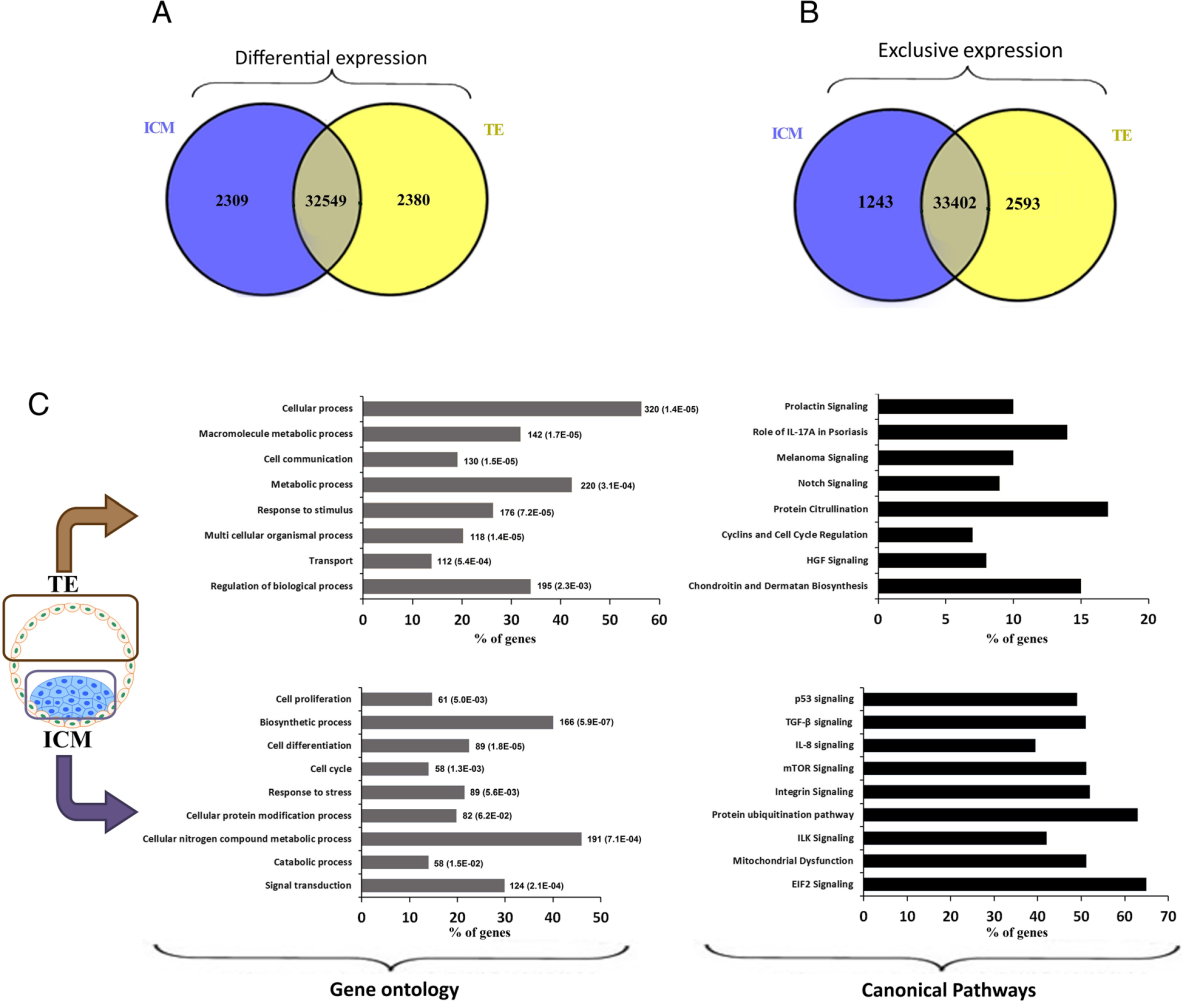
Transcriptome analysis of bovine ICM and TE. **a** Venn diagram indicating the number of genes which differentially expressed between ICM and TE (*P* ≤ 0.01, FC ≥ 2.0, FDR: 2.0). **b** Venn diagram indicating the number of probes with an intensity signal higher than the summation of background intensity and two times its SD for each condition. **c** Gene ontology and canonical pathway analyses of ICM and TE -predominant genes. The number of genes annotated to a GO term and their P value (numbers within the brackets) were shown ahead of the bars

The Circos plot in Fig. 1 provides a genome-wide view on the transcriptome profile of bovine ICM and TE tissues derived from in vivo developed blastocysts. Chromosome mapping of the 100 most differentially expressed genes [green (upregulated) and red (downregulated) overlays in the *p*-value layer of Fig. 1] shows an overall even distribution of differentially expressed genes (DEG) within the bovine blastocyst genome. Significance analysis of microarray data revealed that a total of 4,689 probes were differentially expressed between ICM and TE (Fig. 2a, adjusted P ≤ 0.01, FC ≥ 2.0, FDR = 2.0), which is equal to 12.6 % of all probes assessed. Among these, 2,309 probes were upregulated in ICM, whereas 2,380 probes were downregulated in ICM (i.e. upregulated in TE), respectively (Fig. 2a, Additional file 1: Table S2). Out of the 37,238 targeted probes represented on the microarray slide, a total of 33402 (89.7 %) were present in both ICM and TE tissues, whereas 1,243 (3.4 %) and 2,593 (6.9 %) were exclusively present in ICM and TE tissues, respectively (Fig. 2b, Additional file 1: Table S3).

Ontological classification of the genes predominantly expressed in ICM (Fig. 2c) emerged a range of functional categories with a preponderance of genes involved in cellular nitrogen compound metabolic process, signal transduction, biosynthetic process and cell differentiation. Gene ontology (GO) analysis of TE predominant transcripts (Fig. 2c) emerged a different list of biological processes including the regulation of cellular, biological, and metabolic processes. The most important canonical pathways associated with ICM-predominant transcripts were basal and developmental signaling pathways including P53, TGFβ, IL8, mTOR, integrin, ILK, and ELF2 as well as protein ubiquitination pathway and mitochondrial dysfunction. The most important canonical pathways associated with TE-predominant transcripts were prolactin signaling, melanoma, NOTCH, and HGF -signaling, and protein citrullination (Fig 2c).

**Fig. 3:**
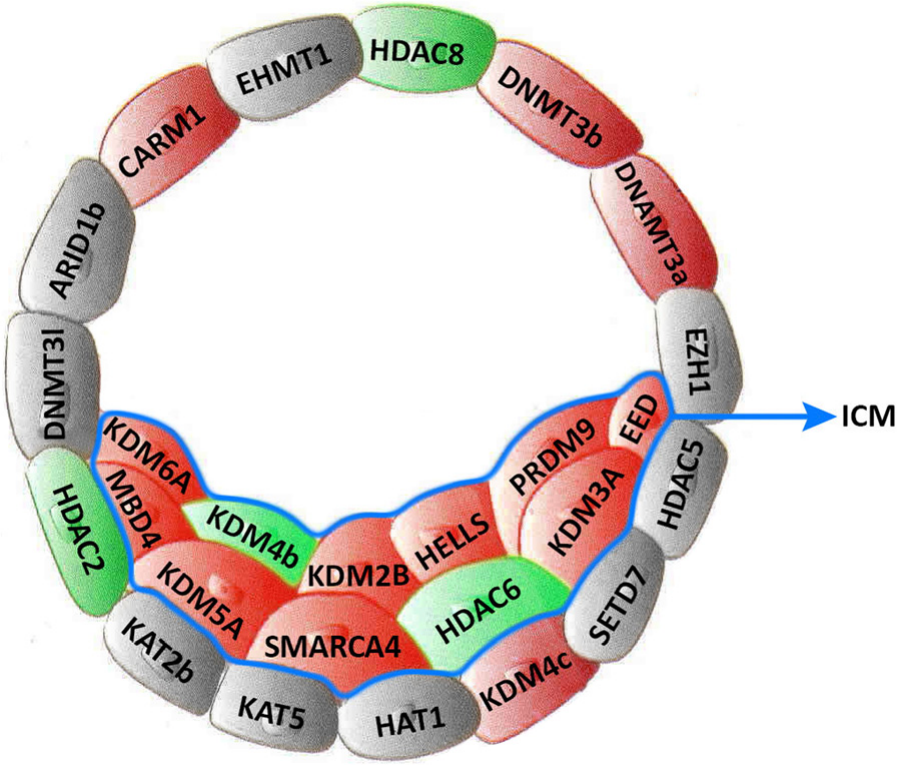
Epigenetic landscape of ICM and TE transcriptomes. **a** Representative image of genes involved in stimulation (ICM-located genes) and repression (TE-located genes) of pluripotency. Red and green cells indicate upregulated and downregulated genes, respectively

Considering the principal role of epigenetic regulation in the process of ICM and TE specification, we profiled the expression of 129 known key epigenetic regulators of pluripotency and differentiation [14] (Additional file 1: Table S4). Of these, 11 transcripts were significantly upregulated in ICM, whereas 6 transcripts were significantly upregulated in TE (Fig. 3). The ICM-upregulated gene list encompassed known genes which were involved in transcriptional activation (KDM3A, SMARCA4, PRDM9, and KDM6A) or transcription repression (HELLS, EED, KDM2B, MBD4, HDAC8, KDM5A, and HDAC2). In contrast, the majority of TE upregulated epigenetic regulators were linked to transcription repression including DNMT3A, DNMT3B, CARM1, and HDAC6 or transcription activation (KDM4B and KDM4C).

### Effect of in vitro culture on transcriptome profile of ICM vs. TE

**Fig. 4:**
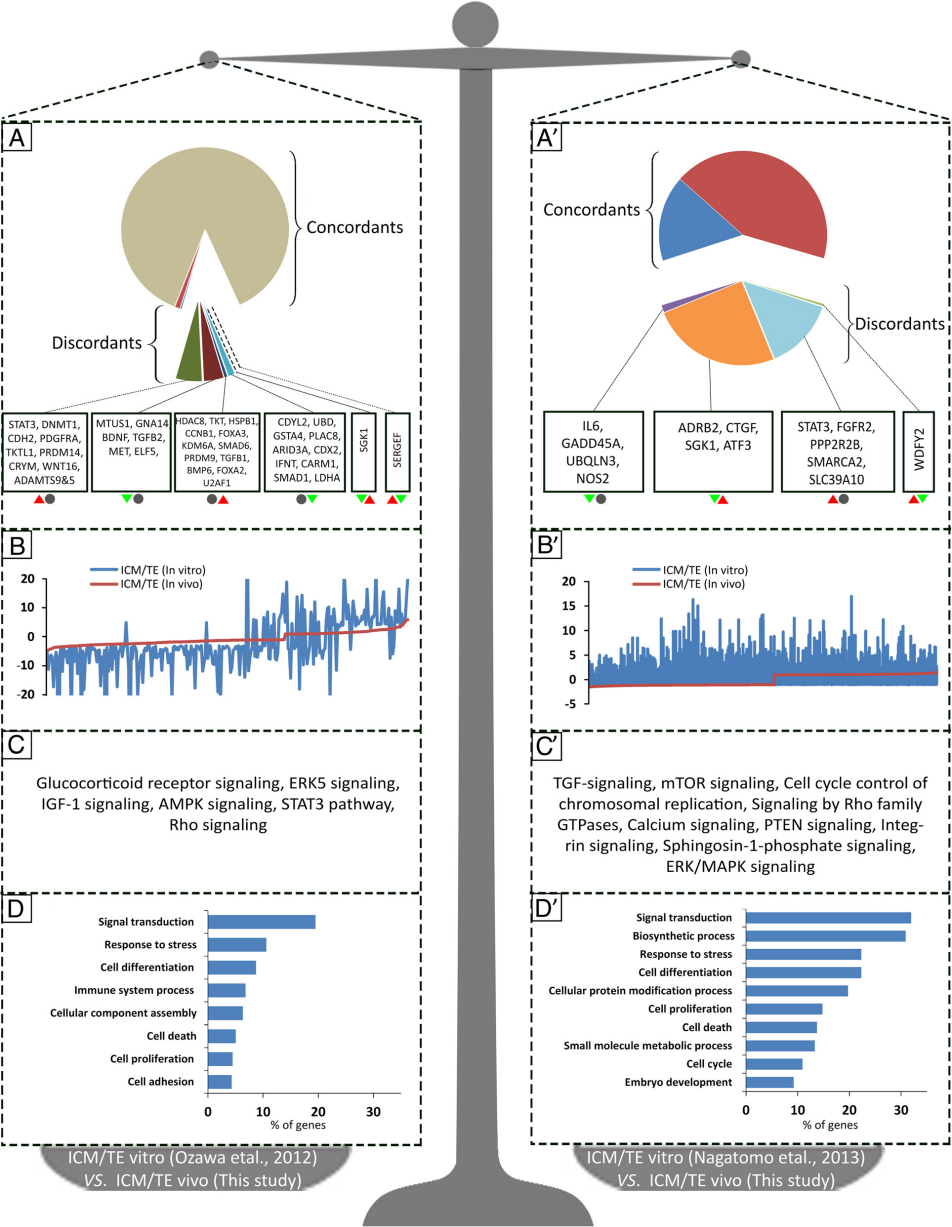
Comparative transcriptome of ICM and TE obtained from in vitro vs. in vivo blastocysts. Transcriptome profile of ICM and TE derived from in vivo generated blastocysts in this study (VIVO) was compared with the in vitro (VITRO) studies of Ozawa et al. [2012] (left panel) and Nagatomo et al. [20] (right panel). A and A’) differential expression with list of important genes misregulated in vitro studies compared to our in vivo study. The colored symbols below the genes lists represent genes that were upregulated (red upward triangle), downregulated (green downward triangle) or similarly (gray circles) expressed in the ICM of vitro (left symbols) vs. VIVO (right symbols) transcriptome studies. B and B’) The overlap between transcriptional profile of in vitro (blue lines) and in vivo (red lines). C and C’) The most important canonical pathways emerged from genes found to be misregulated in ICM and TE transcriptomes of vitro vs. in vivo studies. D and D’) Gene ontology analysis of from genes found to be misregulated in ICM and TE transcriptomes of vitro vs. in vivo studies

To understand the effect of in vitro culture on transcriptional wiring of lineage specification in bovine blastocysts, transcriptome profile of ICM and TE derived from in vivo generated blastocysts (VIVO) was compared with the in vitro studies of Ozawa et al. [6] (VITRO-1) and Nagatomo et al. [7] (VITRO-2). As shown in Additional file 1: Table S5, the total numbers of genes that were differentially expressed were 870 “VITRO-1“and 440 “VITRO-2“which both are substantially lower than 4698 DEGs detected in our study. Specifically, the percentages of genes in “VITRO-1” and “VITRO-2” studies that showed similar expression to the “VIVO” were 90 and 60 %, respectively (concordant plots in Fig. 4A and A’). Moreover, the numbers of genes that were oppositely expressed (i.e. upregulated in “VITRO” but downregulated in “VIVO” or vice versa) were as low as 2 and 5 for VITRO-1 vs. VIVO and VITRO-2 vs. VIVO comparisons (discordant plots in Fig. 4A and A’). Even though, the majority of misregulation found between “VITRO” vs. “VIVO” comparisons was related to genes whose expressions were significant in one VITRO study but not in the counterpart “VIVO” study, and vice versa (Additional file 1: Table S5 and Fig. 4A-B’). This latter category of genes encompasses important gens including STAT3, DNMT1, CARM1, LDHA, GADD45A, FGFR2, IL6, TGFB2, BDNF, ELF5, HDAC8, CCNB1, KDM6A, PRDM9, FOXA3, CDX2, ARID3A, SMAD1, PPP2R2B, SMARCA2, and SLC39A10 (Fig. 4A-B’).

The most important canonical pathways associated with the misregulated genes in “VITRO-1” vs. “VIVO” comparison were ERK5, IGF-1, AMPK, and STAT3 signalings (Fig. 4C). In the same way, canonical pathways associated with misregulated genes in “VITRO-2” vs. “VIVO” comparison belongs to TGFβ, mTOR, cell cycle control of chromosomal, PTEN and ERK-MAPK signalings (Fig. 4C’). Ontological classification of misregulated genes between “VITRO-1” or “VITRO-2” vs. “VIVO” comparisons emerged a range of functional categories with a preponderance of genes required for signal transduction, response to stress and cell differentiation (Fig. 4D and D’).

### Evolutionary perspectives of ICM and TE segregation

**Fig. 5:**
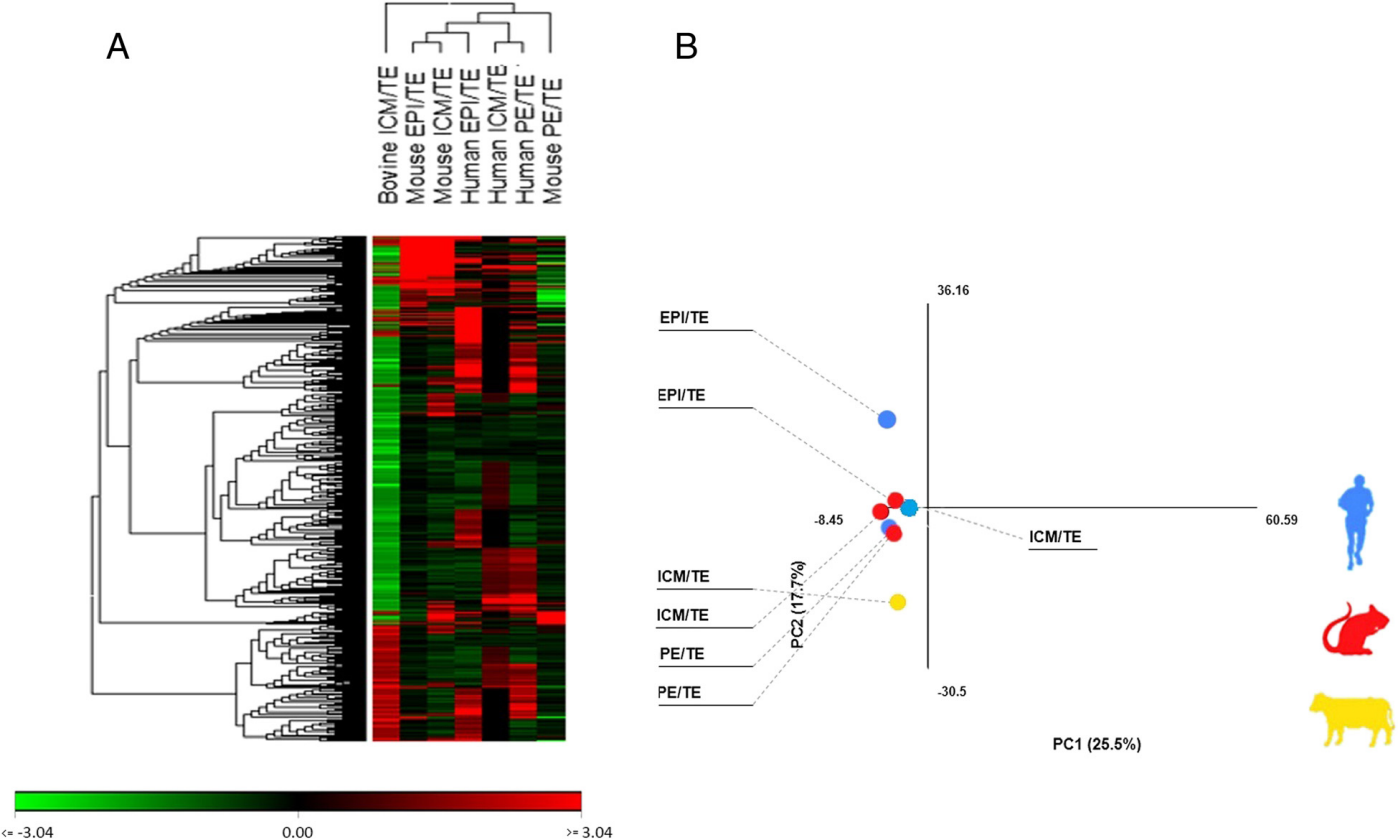
Evolutionary perspective of ICM and TE specification. Cross-species referencing of bovine ICM microarray data obtained in this study with transcriptional data of human and mouse pluripotent cells. Hierarchal clustering (**a**) and principal component analysis (**b**) largely clustered mouse and human ICMs together and separated them from bovine ICM

One intriguing question on the evolution of mammalian species is whether GRNs governing lineage specification and pluripotency are conserved or rewired across species [14, 15]. Accordingly, we contrasted our array data with the transcriptome data of ICM, TE, EPI, and PE tissues obtained from two recent studies in mouse [15] and human [16]. To this aim, we prepared a list of DEGs using those genes that have orthologs in the three species. To visualize the evolutionary relationship between the three species, we performed hierarchical clustering and principal component analysis (PCA) between pluripotent cells of these three species (Fig. 5a and b and Additional file 1: Table S6). The data largely clustered mouse and human ICMs together and separated them from bovine ICM. Since the relative distance between the groups represents the extent of changes in the transcriptome, the molecular signatures of mouse and human ICM share more similarities than bovine. Moreover, bovine ICM clustered away from mouse and human EPI, and PE at the transcriptome level.

**Fig. 6:**
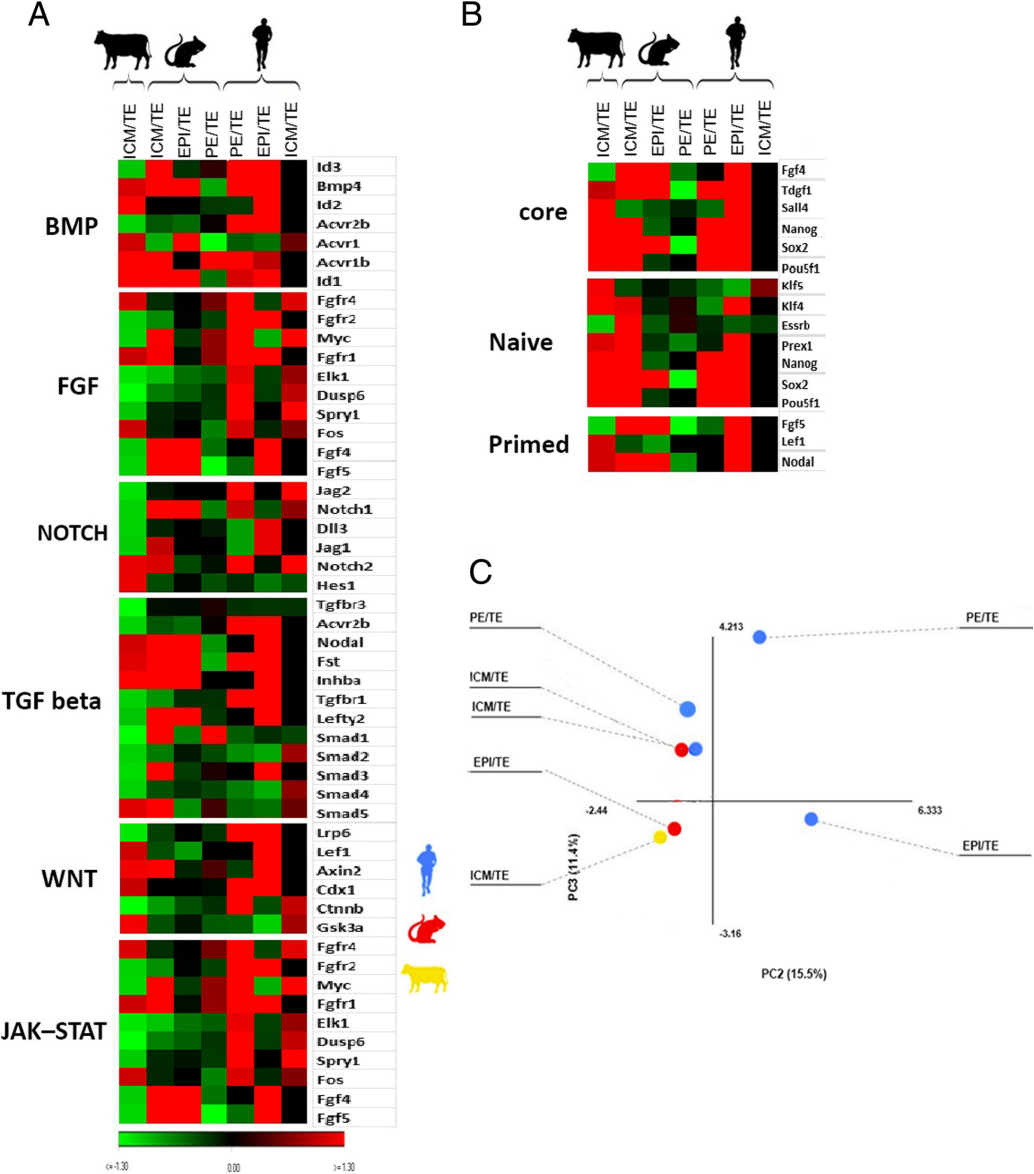
Evolutionary perspectives of gene regulatory networks of pluripotency and differentiation. Cross-species transcriptional profiling of major signaling pathways (**a**) and different pluripotent states of cells (**b**) along with principal component analysis of all genes (**c**)

To validate the assay, we compared the expression of well-established lineage marker genes according to the study of Boroviak et al. [17] (Fig. 6). As shown, the expression profile of key genes that are involved in BMP, FGF, Notch, TGF beta, WNT, and JAK-STAT signaling pathways (Fig 6a) and also in naïve, core and primed states of pluripotency (Fig 6b) were distinct between human, mouse and bovine. To visualize the relationship between the three species with respect to the cumulative genes involved in pluripotency signaling pathways and naïve/core/primed states of pluripotency, we performed principal component analysis (Fig. 6c). The data clustered bovine ICM/TE close to the mouse EPI/TE but away from both human and mouse ICM/TE.

### Microarray validation by RT-qPCR

**Fig. 7:**
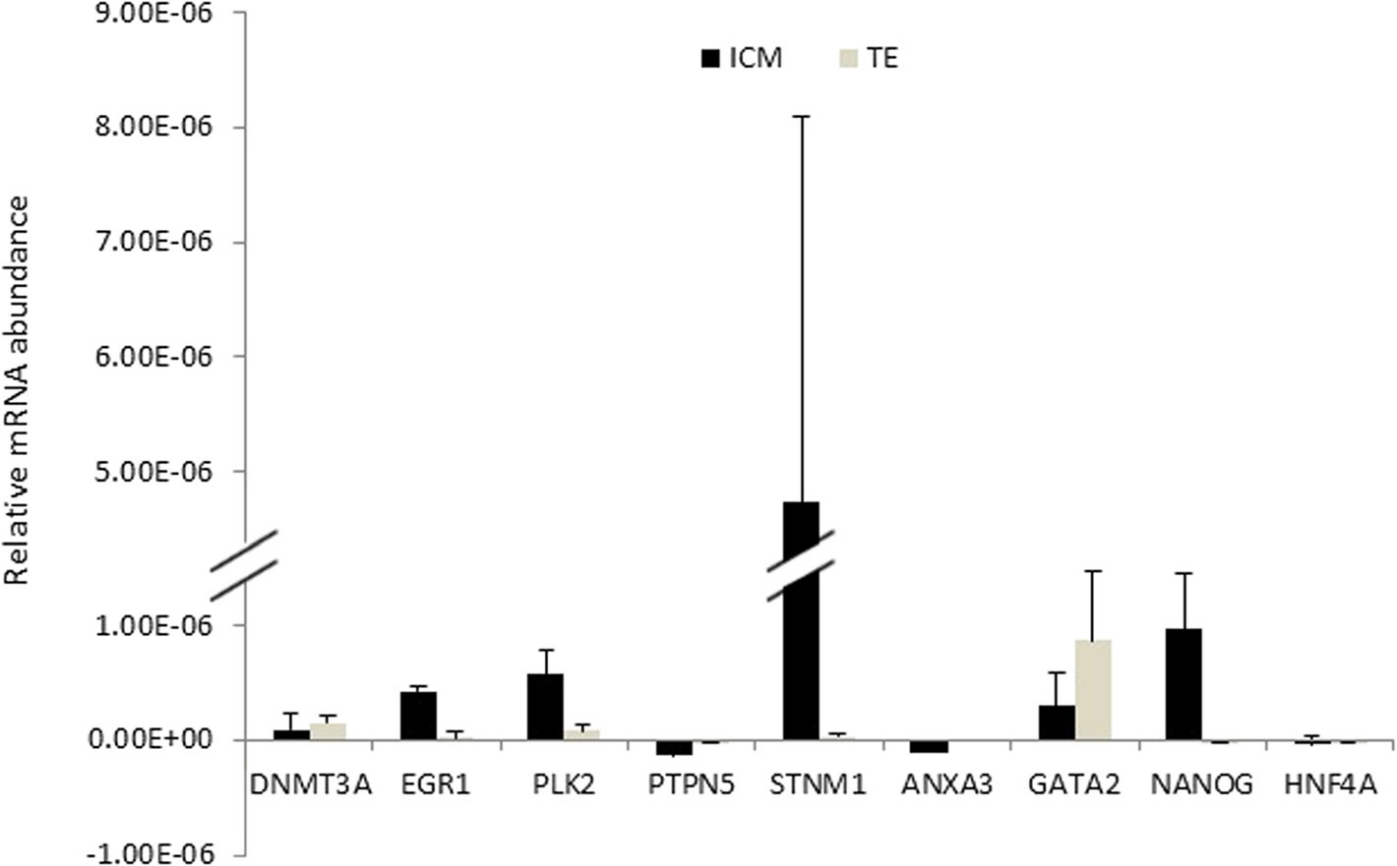
RT-qPCR validation of microarray results. Analysis was done in triplicate and the amount of mRNA represents the mean ± SEM of each transcript corrected with three housekeeping genes. * = *P* < 0.05

To validate the microarray results, RT-qPCR was carried out to investigate the expression levels in ICM and TE of nine genes (DNMT3A, EGR1, PLK2, PTPN5, STMN1, ANXA3, NANOG, HNF4a and GATA2). RT-qPCR was performed in three replicates using mRNAs remained from the same samples used for microarray. The profiles were consistent with the microarray results (Fig. 7), and the expression levels of EGR1, PLK2, NANOG and STMN were higher in ICM than in TE, while expression levels of ANXA3, PTPN5 and GATA2 were higher in TE than in ICM. However, the expression of DNMT3A and HNF4a were inconsistent with microarray results. While DNMT3A was downregulated and HNF4A was upregulated in ICM in the microarray data, RT-qPCR showed no difference between their ICM vs. TE expression. Notably, when we repeated the RT-qPCR of DNMT3A with ICM and TE tissues obtained from in vitro blastocysts, the same trend of DNMT3A downregulation in ICM compared to TE was observed (data not shown).

## Discussion

The first point highlighted in this study is the potential further transcriptional changes between ICM and TE tissues in an in vivo context compared to in vitro. As the in vivo embryos represent the control situation, the first conclusion is that in vitro culture reduces or slows down the differences in directions between the 2 cell types. Accordingly, in vivo-derived ICM and TE showed greater transcriptional differences while counterpart in vitro studies of Ozawa et al. [6] and Nagatomo et al. [7] showed smaller transcriptional difference, suggesting that in vitro culture may reduce the actual transcriptional differences of bovine ICM and TE specification. Another possible explanation is that the in vitro developed blastocysts may be just delayed compared to their age-matched in vivo counterparts. Therefore, the divergence between the ICM and TE lineage would be less pronounced but due to this delayed timing. In agreement with our results, Guo et al. [18] and Giritharan, et al. [12] demonstrated that while ICM and TE from mouse in vivo blastocyst showed many differences in transcriptome, their in vitro counterparts had a muted level of differentiation between ICM and TE. Importantly, a great majority of genes that were found to be misregulated following in vitro culture of bovine embryos were known genes involved in epigenetic regulation of pluripotency and cell differentiation including DNMT1, GADD45, CARM1, ELF5 HDAC8, CCNB1, KDM6A, PRDM9, CDX2, ARID3A, IL6, GADD45A, FGFR2, PPP2R2B, and SMARCA2 [14].

The second interesting and new observation is the discriminative cross-species differences between transcriptional profile of bovine ICM and other species. For example, CDX2 transcripts were detected in both ICM and TE, which disagrees with what is confirmed in human and mouse [5, 19]. This observation is even inconsistent with bovine studies that demonstrated CDX2 exclusive expression in TE by different approaches including immunostaining [7, 20], microarray [7] and RNA-sequencing [6]. However, our result is in agreement with Berg et al. [20] who also showed continuous expression of CDX2 in both tissues in bovine day 7-11 embryos. On the other hand, TEAD4 and GATA4 were exclusively expressed in bovine ICM. Kuijk et al. [5] detected GATA4 protein in both ICM and TE, and restricted expression of GATA6 in bovine ICM. We also observed exclusive expression of GATA4 transcripts in ICM and expression of GATA6 in both ICM and TE tissues. Although the exact reason of this discrepancy is not understood, it could be due to the time lapse between transcription and translation of mRNA. We measured mRNAs, which may be translated several hours later [21, 22, 23]; therefore, the proteins are probably needed and presented at different times than the mRNAs.

The third new observation was substantial divergence between human, mouse and bovine in signaling pathways involved in early lineage specification. Since the chronological sequence of embryonic events leading to the segregation of ICM from TE is thought to be similar across mammals [24], it is possible that the “timing” and/or the “mode” of interaction between key developmental regulators of early lineage specification rather diverged from the ancestral model. Accordingly, embryos of each species develop unique features that may make them different from closely related species. In line with the “timing” hypothesis, mouse embryos implant very soon after blastocyst formation in embryonic day (ED) 3.5 when the ICM and TE fates become firmly sealed by reciprocal expression of OCT4 and CDX2 in the ICM and TE tissues, respectively [25]. Accordingly, functional repression of OCT4 produces blastocysts lacking a pluripotent ICM because of dominant differentiation of ICM cells into extra-embryonic trophoblast fate, even if CDX2 was ablated in such an embryo [26]. In same way, CDX2^-/-^ mice embryos fail to develop to blastocysts due to the lack of TE integrity and blastocoel collapse [25]. In human embryos that implant later than mouse in ED5, OCT4 is still detected in both ICM and TE tissues at this stage. Moreover, it has been demonstrated that primate ESC is more equivalent to mouse EPI-stem cells, which are driven from post implantation embryos, and is developmentally advanced relative to mouse naive ESC [27]. We also observed that bovine ICM is closer to mouse EPI when looking at pluripotency, signaling and lineage genes. Implantation in bovine, unlike human and mice, occurs with a delay of around 7 days after blastocyst formation [7]. Importantly, by reciprocal exchange of the mouse and bovine OCT4 promoter, Berg et al. [20] provided unequivocal evidence that bovine TE is yet not committed at equivalent stages to the mouse blastocyst. This may explain why bovine embryos depleted from CDX2 mRNA could develop to the blastocyst stage without apparent abnormality in the ICM and TE cell counts [28]. In line with the “mode” of interaction hypothesis, several recent studies demonstrated that developing mammalian embryos have intrinsic differences in their response to a given signal transduction pathway. For example, inhibition of MEK, the nuclear effector of FGF signaling pathway, completely ablates PE formation in mouse embryos, but only partially interferes with PE in bovine embryos, and does not interfere with the PE formation in human embryos [2].

An improved understanding of gene activity that regulates preimplantation development in each species provided clues for improvement of assisted reproduction techniques and for derivation of embryonic stem cells [24, 25]. For example, Khan et al. [29] introduced SOX2 and NANOG, rather than OCT4, as the possible earlier candidates of ICM specification and hence pertinent markers of pluripotent lineage specification in bovine. Harris et al. [24] showed that simultaneous inhibition of the FGF and stimulating the WNT signalings accelerated blastocyst development and increased ICM and TE by counts in bovine. McLean et al. [30] showed that chemical inhibition of FGF and WNT pathways during in vitro culture of bovine embryos specifically promoted a shift from a hypoblast to an epiblast gene expression signature in ICM cells. Using the same treatment of ICM cells isolated from in vitro developed bovine blastocyst, Verma et al. [31] could prime ICM cells for stem cell derivation through sustained expression of SOX2 and NANOG (EPI-markers), while repressing GATA4 (PE-markers) during continuous culture.

There is evidence for a transcriptional switch from pyruvate to glucose dependency during the transition from early-to-late genome activation in several species [24, 32]. However, the contribution made by the ICM and the TE to the overall energy metabolism of intact blastocysts has received relatively little attention [33]. Accordingly, the other interesting point revealed in this study was that totally different metabolic pathways are operating in the ICM and TE tissues in bovine. For example, LDHA, a gene coding for the main enzyme of anaerobic conversion of pyruvate into lactate [34], was downregulated; while TKT1, a gene coding an enzyme that catalyzes the non-oxidative part of the pentose phosphate pathway, and HNF4a were upregulated in ICM, suggesting suppression of glycolysis, but stimulation of the pentose phosphate and tricarboxcy acid cycle shunts in ICM compared to TE. This is also different from the metabolic pathway that is frequently observed in ESCs: the “Warburg effect”, a metabolic shift from mitochondrial oxidation-phosphorylation to glycolysis. Further confirmation on different metabolic pathways in ICM and ESC comes from the observation that PDGFC and HIF1a, which contribute to the expression of LDHA [35] and promote anaerobic glycolysis in tumor cells [36, 37], were downregulated in bovine ICM cells. Our conclusion that different metabolic pathways are operating in bovine ICM and TE is consistent with the study of Houghton et al. [33] on metabolic profiling of mouse ICM and TE. Genes involved in cholesterol metabolism (HSD3B1, HSD17B1, and FDX1), estrogen biosynthesis (CYP11A1, CYP19A1), and lipid metabolism (PTGES) were all upregulated in TE, which is in agreement with Assou et al. [32] who suggested a central role of these steroidogenic enzymes in the steroid biosynthesis and metabolism of TE. Thus, one may argue that bovine (in this study) and human TE [32] are steroidogenically active tissues similar to the brain, heart, gonads, endometrium, and placenta [38, 39].

Epigenetic reprogramming is highly dynamic during embryogenesis, particularly during the first lineage commitment [59]. Human ICM has been recognized hypomethylated [40]. Mouse ICM has been reported hypomethylated just before the onset of de novo methylation [41], although de novo methylation is specifically observed in the mouse ICM but not TE [42]. Bovine ICM was also reported hypomethylated [43, 44] which is consistent with our microarray results. On the other hand, DNMT3A is specifically expressed in EPI and ESC cells in the mouse [45]. DNMT3A and DNMT3B are both enriched in human ESC [40] and human ICM [1]. In bovine, however, we observed that DNMT3A and DNMT3B were upregulated in TE by 2.2 fold compared to ICM (Fig. 3). Notably, the expression of TET1 and TET2, which are critical modulators of DNA demethylation in ESC, was not different between bovine ICM and TE, whereas they are highly expressed in mouse ICM and ESCs [14]. We deduced that these species-specific differences in expression of DNMT3A and DNMT3B and TET1 and TET2 may provide clues for epigenetic engineering of bovine ICM toward ESC derivation. Investigation of the epigenomes of bovine ICM and TE is required to achieve this goal.

## Conclusion

This study provided a standard to evaluate the in vitro imprint on the transcriptome. Using this standard, we showed that the ICM vs. TE transcriptional difference reduces following in vitro culture of bovine embryos. We also showed that mouse and human ICMs are separated from bovine ICM at the transcriptome level. These results provided better understanding of the cross-species differences that allow in vitro acquisition of pluripotent ICM in human and mice but hinder that process in bovine.

## Methods

All reagents were obtained from Sigma-Aldrich (St-Louis, MO, USA) unless otherwise specified.

### In vivo embryo production and embryo collection

Animals were cared for according to the recommended code of practice following the guidelines of the Canadian Council on Animal Care (1993). Production of in vivo embryos was carried out using 26 superovulated Holstein cows and semen prepared from seven bulls as described previously [46]. The content of the dominant follicles (>6 mm) was aspirated using an ultrasound guided system 36–48 h before the first FSH injection; this was any time between day 6 and day 12 of the estrous cycle. A total of 320 mg p-FSH (Folltropin; Bioniche Animal Health, Montréal, Quebec, Canada) was given in eight IM injections of decreasing concentrations over a 5-day period. A prostaglandin injection (500 μg Estrumate; Schering, Montréal, Quebec, Canada) was given to induce luteolysis of the corpus luteum at the eighth p-FSH injection. Another prostaglandin injection was given 12 h after the first one. The cows were inseminated with frozen semen 12 and 24 h after the first signs of estrus. The embryos were recovered at day 7.5 post insemination by flushing the uterine horns with phosphate buffer saline (PBS). Blastocysts of good morphological quality were washed twice in PBS, and transported to the laboratory for ICM and TE separation. The superovulation treatment resulted in 4.9 ± 0.9 ovulations per cow and 2.0 ± 0.4 embryos recovered per flush. A total of 277 embryos were retrieved by flushing at day 7.5 post insemination. Of these, 174 were expanded blastocysts of good quality. Four separate pools of ICM and TE tissues were obtained using these selected blastocysts (Additional file 1: Table S1). Each pool was used for one microarray replicate.

### Preparation of ICM and TE

Good quality expanded blastocysts were used for ICM and TE separation. In brief, blastocysts were first incubated in acid Tyrode’s for one min to digest the zona pellucida followed by washing and incubating in PBS to dilute and stop the reaction. Tissues were isolated by microsurgery and immunosurgery. In brief, blastocysts were first cut into pure TE and ICM-TE halves with a micro scalpel (Bioniche, Ultra Sharp Splitting blades, ESE020). The ICM-TE fragments were incubated in Dulbecco’s modified eagle medium (DMEM; Gibco 11885-076, USA) containing rabbit anti-bovine whole serum (Sigma B8270) in a ratio of 1:15 at 38.5 °C for 1 h followed by repeated washings in PBS. Samples were then incubated in DMEM containing 1/5 (V/V) of guinea pig complement (Rockland, C300-0010) for 45 min at 38.5 °C to lyse TE cells and retrieve pure ICM. Isolated ICM and TE tissues were washed in nuclease-free PBS and stored at −80 °C until use.

### RNA isolation, amplification, and microarray hybridization

The whole process of microarray analysis was as described previously [34]. Total RNA from each replicate of pooled ICM and TE was extracted and purified using the PicoPure RNA Isolation Kit (Life Science). After DNase digestion (Qiagen), the quality and concentration of the extracted RNA were analyzed by bioanalyzer (Agilent). All extracted samples were of good quality, with an RNA integrity number ≥7.0.

Purified total RNA was amplified by in vitro transcription by T7 RNA amplification using the RiboAmp HS^Plus^ RNA Amplification Kit (Life Science), and labeled with Cy3 and Cy5 using the ULS Fluorescent Labeling Kit (Kreatech). Antisense RNA (825 ng per replicate) was hybridized on Agilent-manufactured EmbryoGENE® slides [13] in a two-color dye-swap design. The microarray chip used, EmbryoGENE®, covers almost all of the bovine pre-attachment transcriptome and hence allows the analysis of most forms of gene expression in bovine blastocysts.

After 17 h of hybridization at 65 °C, microarray slides were washed for 1 min in gene expression wash buffer 1 (RT), 3 min in gene expression wash buffer 2 (42 °C), 10 s in 100 % acetonitrile (RT) and 30 s in Stabilization and Drying Solution (Agilent). Slides were scanned with a Power Scanner (Tecan), and features extraction was done with Array-pro6.3 (Media Cybernetics). Intensity files were analyzed with FlexArray 1.6.1 (Michal Blazejczyk, Mathieu Miron, Robert Nadon (2007), FlexArray: statistical data analysis software for gene expression microarrays. Genome Quebec, Montreal, Canada, URL:http://genomequebec.mcgill.ca/FlexArray). Specifically, raw data were corrected by background subtraction, and then normalized within and between each array (Loess and quantile, respectively). Statistical comparison of the treatments was done with the Limma algorithm. Transcripts with a false discovery rate (FDR) of 20 %, a fold change >2.0, and a *p*-value <0.01 were considered differentially expressed. The data discussed in this publication were deposited in NCBI's Gene Expression Omnibus and are accessible through GEO Series accession number (http://www.ncbi.nlm.nih.gov/geo/query/acc.cgi?acc=GSE75555.

#### Quantitative RT-PCR

Validation of microarray results was done by quantitative real-time PCR (RT-qPCR). Total RNA from independent samples (three replicates for each condition) was reverse transcribed using oligo-dT primers and the qScript Flex cDNA Synthesis Kit (Quanta Biosciences). Specific primers for each selected gene were designed using PrimerQuest (Integrated DNA Technologies) and qPCR was performed with the LightCycler 480 SYBR Green I Master and the LightCycler 480 System (Roche). A standard curve constituted of five points of the PCR product for each primer pair, diluted from 1 pg to 0.1 fg, was used for real-time quantification of PCR output. Data were normalized with the GeNORM normalization factor from the expression values of three reference genes. Primer sequences, product sizes, annealing temperatures, and accession numbers are provided in Additional file 1: Table S7.

#### Functional analysis of differential gene expression profiles

The ingenuity Pathways Analysis (IPA; Ingenuity Systems, www.ingenuity.com) software was used to group overrepresented functions of differentially expressed genes into clusters. Moreover, IPA was queried to compile canonical pathways, as well as gene regulatory networks that were differentially expressed between treatments. We used IPA to build schematic representations of important pathways in ICM and TE.

## Additional file

Additional file 1
**Table S1.** Embryo recovery data. Detail information of in vivo embryo production and ICM/TE immunosurgery. Table S2. Differentially regulated gens. List of genes differentially expressed between ICM and TE of in vivo-derived blastocysts (FDR 2, *P* ≤ 0.01, FC ≥2). Table S3. ICM/TE predominant gene list. Breakdown of genes exclusively expressed in ICM and TE after filtering data against background genes. Table S4. Epigenetic landscape of ICM transcriptome. Transcriptional profile of epigenetic genes expressed in ICM compared to TE in bovine. Table S5. Effect of in vitro culture on transcriptional profile of ICM and TE. Comparison between transcriptome data of bovine ICM and TE obtained from in vivo (This study) and in vitro (Ozawa et al. [19], and Nagomoto et al. [20]) studies. Table S6. Comparative transcriptional profile of pluripotency related genes between human, mouse and bovine. Expression profile of genes with similar identity between a human [16] study and a mouse study [14] were compared with this study. Table S7. RT-qPCR primers. Sequences (5’–3’) of reverse transcription qRT-PCR-specific primers of candidate genes expressed in bovine embryos. (XLSX 1729 kb)

## Abbreviations

ICM: inner cell mass
TE: trophectoderm
ESC: embryonic stem cells
PSC: pluripotent stem cells
IPA: Ingenuity Pathways Analysis
PCA: principal component analysis
5mC: 5 methyl cytosine
5hmC: 5 hydroxymethyl cytosine
5fmC: 5 formylmethyl cytosine
DEG: differentially expressed genes
RT-qPCR: quantitative real-time polymerase chain reaction
PBS: phosphate buffer saline
RT: room temperature
